# Investigation of factors contributing to pedestrian crash severity in rural roads

**Published:** 2019-08

**Authors:** Mahyar Vahedi Saheli, Meysam Effati

**Affiliations:** ^*a*^Department of Civil Engineering, Faculty of Engineering, University of Guilan, Rasht, Iran.

## Abstract

**Background:**

More than half of all traffic deaths are among vulnerable road users. Pedestrians are known as the most vulnerable road users to injury and fatality in road crashes. In 2018, the World Health Organization (WHO) reported a pedestrian fatality rate of 4.4 per 100000 people for Iran that is higher than the world average. Pedestrians experience more severe crashes in rural roads due to the higher speed of vehicles. This paper identifies and compares the factors affecting pedestrian crash severity in rural roads.

**Methods:**

A number of 3067 rural road pedestrian crashes data in Guilan province were extracted for the study between the years of 2014-2017 that contains 416 fatal and 2651 Injury crashes. A binary logistic regression is used due to the existence of a binary outcome of crash severity (injury or fatality). Study variables consist of road characteristics (median dividing of the road, curve, grade, lighting condition, and road surface condition), pedestrian and driver characteristics (gender and age), the vehicle involved (automobile, motorcycle, pickups, van/bus/minibus, and heavy vehicle) and hit-and-run crashes. Among these variables, median-divided roads, men and old pedestrians were found to be statistically significant with increasing impact and young, and child pedestrians, young and adult drivers and daylight condition were statistically significant with decreasing impact on pedestrian crash severity.

**Results:**

Results are broadly consistent with previous research and offer some new insights. For example, many studies emphasized on the ageing impact on reaction time for pedestrians; more dangerous behaviors in male pedestrian in comparison with females and more severe crashes in median-divided roads due to higher speeds.

**Conclusions:**

The findings suggest some countermeasures that help policy-makers to reduce pedestrian fatalities.

**Keywords:**

Vulnerable road users, Pedestrian safety, Crash severity, Logistic

